# Levothyroxine therapy, calculated deiodinases activity and basal metabolic rate in obese or nonobese patients after total thyroidectomy for differentiated thyroid cancer, results of a retrospective observational study

**DOI:** 10.1002/edm2.406

**Published:** 2023-02-01

**Authors:** Rosario Le Moli, Pasqualino Malandrino, Marco Russo, Dario Tumino, Tommaso Piticchio, Adriano Naselli, Valentina Rapicavoli, Antonino Belfiore, Francesco Frasca

**Affiliations:** ^1^ Endocrinology Unit, Department of Clinical and Experimental Medicine University of Catania, Garibaldi Nesima Hospital Catania Italy

**Keywords:** deiodination, hypothyroidism, LT‐4 treatment, obesity

## Abstract

**Introduction:**

Therapy for hypothyroid obese patients is still under definition since the thyrotropin‐stimulating hormone (TSH) level is a less reliable marker of euthyroidism than nonobese patients. Indeed, TSH levels positively correlate with body mass index (BMI), and this increase may be a compensatory mechanism aimed at increasing energy expenditure in obese people. In contrast, the correlation of BMI with thyroid hormone levels is not completely clear, and conflicting results have been obtained by several studies. The L‐T4 replacement dose is more variable in obese hypothyroid patients than in nonobese patients, and a recent study indicated that the L‐T4 replacement dose is related to lean body mass in obese thyroidectomized patients. We aimed to study the correlations of L‐T4‐administered dose, thyroid hormone levels and TSH secretion with basal metabolic rate (BMR) and total calculated deiodinase activity (GD) in obese and nonobese athyreotic patients. We also looked for individualized L‐T4 replacement dose set points to be used in clinical practice.

**Methods:**

We studied retrospectively 160 athyreotic patients, 120 nonobese and 40 obese. GD was calculated by SPINA Thyr 4.2, the responsiveness of the hypothalamic/pituitary thyrotrope by Jostel's thyrotropin (TSH) index and BMR by the Mifflin‐St. Jeor formula, the interplay of GD and BMR with L‐T4, thyroid hormones and TSH index (TSHI) was also evaluated.

**Results:**

In our study, the L‐T4 dose was an independent predictor of GD, and approximately 30% of athyreotic patients under L‐T4 therapy had a reduced GD; FT4 levels were higher and negatively modulated by BMR in obese athyreotic patients respect to nonobese, in these patients a T4 to T3 shunt, in terms of TSHI suppression is observed suggesting a defective hypothalamic pituitary T4 to T3 conversion and a resistance to L‐T4 replacement therapy.

**Conclusions:**

L‐t4 dose is the most important predictor of GD, BMR modulates T4 levels in obese athyreotic patients that are resistant to L‐T4 replacement therapy.

## Graphical abstract

Because of controversial issues about the optimal T4 replacement dose in obese hypothyroid subjects and the great importance of thyroid hormones in energy homeostasis, glucose and lipid metabolism, body composition and resting energy expenditure (REE), we compared the correlation between L‐T4 administered dose, thyroid hormone levels and TSH secretion with estimated basal metabolic rate (BMR) and total deiodinase activity (GD) in obese and nonobese athyreotic subjects. We aimed to define individualized set points that might provide appropriate therapeutic and biochemical targets to be clinically tested in obese and nonobese patients.

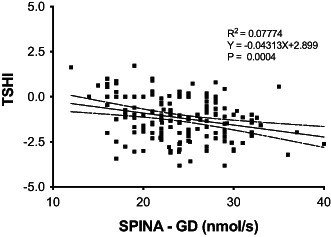

## 1 INTRODUCTION

Levothyroxine (L‐T4) therapy has a long record in clinical use, with a defined pharmacological profile and safety in hypothyroidism management. Obesity and thyroid disorders are common among the general population and may be associated with both clinical and molecular aspects. This relationship has become epidemiologically relevant in the context of the significantly increased prevalence of obesity worldwide. However, treatment for obese patients with subclinical or overt hypothyroidism is still under definition regarding both the threshold and modality (liquid L‐T4 vs. pills; L‐T4 monotherapy vs. liothyronine [L‐T3]/L‐T4 combinations). The prerequisite for treatment with L‐T4 is the presence of hypothyroidism, and the goal is restoration of euthyroidism. Achievement of a thyrotropin‐stimulating hormone (TSH) value within the age‐adjusted euthyroid range is the accepted therapeutic target, as several studies indicate improvement in symptoms, quality of life and cardiovascular risk.^1^, ^2^, ^3^, ^4^ However, among euthyroid subjects, TSH levels usually correlate with body mass index (BMI), being higher in obese than in normal subjects.^5^ TSH elevation in obese euthyroid people may be a compensatory mechanism in the pituitary‐thyroid axis aimed at increasing energy expenditure.^6^, ^7^ At variance with TSH, the correlation between BMI and thyroid hormones (T4 and T3) is not clear, as several studies obtained conflicting results. Some studies indicate that BMI is negatively related to FT4 and positively related to FT3. Another study, by contrast, indicated hyperactivation of the pituitary‐thyroid axis with increased FT4 levels in obese patients.^8^, ^9^ Other studies describe a decreased FT4/FT3 ratio in obese patients.^5^, ^10^, ^11^, ^12^ This adaptation of thyroid hormone homeostasis in obese subjects has been attributed to leptin and insulin actions.^3^ The observation of higher TSH and lower FT4 in obese euthyroid people is in accordance with increased L‐thyroxine replacement dose in hypothyroid obese patients. L‐T4 replacement therapy is approximately 1.6 μg/kg in hypothyroid patients with any functional thyroid tissue, while in obese patients, the correct T4 replacement dose is more variable. Recently, the American Thyroid Association (ATA) task force identified obesity as a morbid condition implying an increase in the L‐T4 replacement dose because of reduced thyroid hormone absorption.^2^ This observation is reinforced by the evidence that in obese subjects, acute overload of L‐T4 administration takes longer to achieve a plasmatic concentration peak in comparison with nonobese people.^1^ However, a recent study indicated that in obese thyroidectomized patients, the L‐T4 replacement dose is positively related to lean body mass. Indeed, the ideal body weight (IBW) should be preferred to real body weight (RBW) for L‐T4 dose titration because lean body mass results in a better predictor of T4 requirement than fat mass.^6^, ^7^ Because of these controversial issues about the optimal T4 replacement dose in obese hypothyroid subjects and the great importance of thyroid hormones in energy homeostasis, glucose and lipid metabolism, body composition and resting energy expenditure (REE),^10^, ^12^, ^13^, ^14^ we compared the correlation between L‐T4‐administered dose, thyroid hormone levels and TSH secretion with estimated basal metabolic rate (BMR) and total deiodinase activity (GD) in obese and nonobese athyreotic subjects. Moreover, we aimed to define individualized set points that might provide appropriate therapeutic and biochemical targets to be clinically tested in obese and nonobese patients.

## 2 PATIENTS AND METHODS

### 2.1 Patients

**FIGURE 1:**
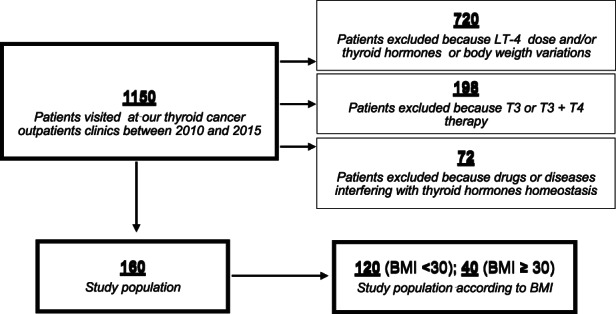
Flow chart of study patients selection

We retrospectively evaluated 1150 thyroidectomized patients referred to our outpatient thyroid clinic between 2010 and 2015 who were also subjected to ^131^I ablation because of differentiated thyroid cancer (DTC). In all patients, thyroglobulin levels were between 0.01 and 0.5 ng/ml, and antithyroglobulin antibody (TgAb) was negative. In this cohort, devoid of functional thyroid tissue, all circulating T4 levels originated from levothyroxine replacement therapy. These patients obtain circulating T3 from the conversion of exogenous T4 and represent an ideal model to study peripheral tissue ability to generate biologically active hormones. We excluded from the analysis patients with hypothalamic/pituitary, gastric, intestinal or neurological diseases and pregnant women (*n* = 72) and those who were taking combined T3/T4 thyroid replacement therapy and/or other drugs interfering with thyroid hormone homeostasis (*n* = 198). Patients with variations in L‐T4 daily dose, body weight and thyroid hormone level fluctuations within 3 months before the start of the study were also excluded (*n* = 720). Finally, 160 athyreotic patients under L‐T4 therapy were included in the analysis (Figure 1). All patients were euthyroid on the basis of their TSH, FT4 and FT3 levels within the normal range.

### 2.2 Phenotypic evaluation of the study patients

Clinical records included a detailed history, physical examination, standardized questionnaire documenting sex, age, height, weight and BMI. BMI was calculated as weight in kilograms divided by the square of height in metres (kg/m^2^) and considered a categorical variable according to the World Health Organization (WHO). Obesity was defined as BMI ≥ 30, which is an adequate indicator of obesity and is associated with increased body fat mass. In our study, 120 patients had a BMI ≥ 30, while 40 had a BMI < 30.

### 2.3 Basal metabolic rate (BMR) evaluation

We evaluated BMR by the Mifflin‐St. Jeor formula (MSTF). The MSTF equation is commonly used in the assessment of basal metabolism and is more particularly used in obese patients. The MSTF was also applied differently to female and male sex as follows:

- Females = 9.99 × weight (kg) + 6.25 × height (cm) − 4.92 × age (years) − 161.
- Males = 9.99 × weight (kg) + 6.25 × height (cm) − 4.92 × age (years) + 5.

We studied the effect of the L‐T4 replacement dose on thyroid hormone homeostasis, estimated BMR and total deiodinase activity (GD) in obese and nonobese patients. Data were collected from patients after thyroidectomy, ^131^I administration and a persistent euthyroid state under replacement therapy for approximately 3 months with any significant change in L‐T4 dose administration, daily caloric intake and body weight. A subgroup of 45 patients maintaining the same replacement dose over the last 6 months was also studied to better evaluate the interplay between the L‐T4 administered dose and total GD in the long term.

### 2.4 Evaluation of stimulated deiodination (GD)

GD, which reflects the maximum stimulated activity of deiodination, was calculated by SPINA Thyr 4.2 (Structure Parameter Inference Approach by Johannes W. Dietrich, Lab XU44, Bergmannsheil University Hospitals, Ruhr University of Bochum, D‐44789 Bochum, NRW, Germany), which is a mathematical tool for the integrated interpretation of laboratory results. SPINA allows calculation of GD from TSH, FT4 and FT3 serum levels obtained from routine laboratory assays. The method is based upon mathematical/cybernetic modelling of processing structures.^15^ In particular, the SPINA algorithm is based on equilibrium analysis of a compartmental nonlinear model: GD = $\frac{\beta 31\left(\mathrm{Km}1+\left[\text{FT}4\right]\right)\left(1+K30\left[\mathrm{TBG}\right]\right)\left[\text{FT}3\right]}{\alpha 31\left[\text{FT}4\right]}$, where β31 is the clearance exponent for T3, *Km*1 is the dissociation constant of type 1 deiodinase, *K*30 is the dissociation constant of T3 at thyroxine‐binding globulin, and α31 is the dilution factor for triiodothyronine. On the basis of several studies, normal values of calculated GD vary between 21 and 26 nmol/s.^15^, ^16^ Hence, a GD < 21 nmol/s is considered low.

### 2.5 Responsiveness of the hypothalamic/pituitary thyrotrope

We also assessed the responsiveness of the hypothalamic/pituitary thyrotrope by Jostel's thyrotropin (TSH) index: (JTSHI) = ln([TSH]) + β[FT4] and obtained a standardized TSH index (TSHI) = JTSHI − 2.7/0.676 for statistical comparison.

### 2.6 Laboratory measurements

Serum TSH was assessed by an ultrasensitive enhanced chemiluminescence immunoassay (ECLIA) assay. Serum hormones were measured by microparticle enzyme immunoassay (Abbot AxSYM‐MEIA) with interassay coefficients of variation of less than 10% over the analytical ranges of 1.7–46.0 pmol/L for FT3, 5.15–77.0 pmol/L for FT4 and 0.03–10.0 mU/L for TSH. The within‐run and between‐run precisions for the FT3, FT4 and TSH assays showed coefficients of variation <5%. Measurement of antithyroglobulin antibodies (TgAbs) by an automated chemiluminescence assay system (AntiTg, Ready Pack). Thyroglobulin levels were measured with a second‐generation chemiluminescent Tg immunoassay (Tg Access; Beckman Coulter) with a functional sensitivity of 0.1 ng/ml.

### 2.7 Statistical analysis

Statistical analysis was performed using the SPSS package (IBM SPSS Statistics for Windows, Version 26.0. IBM Corp). For the descriptive analysis, continuous variables were expressed as the mean ± standard deviation (SD) or median (with its 25th–75th percentile); categorical variables were expressed as numbers and percentages. Univariate analysis of variance (ANOVA) was performed to identify predictive variables significantly associated with the clinical outcome. The shapes of the distribution of each variable were evaluated by visual inspection of the population pyramid charts; for distributions of similar shapes, we reported the medians, and for distributions of different shapes, we reported average ranks. The Mann–Whitney *U* test was used to analyse the continuous variables without a normal distribution. Categorical variables were analysed by the Chi‐square test, if cells with fewer than five expected cell numbers were found, by Fisher's exact test. Complete and partial bivariate analysis was used to evaluate no categorical variables, and Pearson's coefficient was computed. Binary logistic regression analysis was performed for the outcome variables. Covariates were selected on the basis of the results of univariate analysis, and the final model was built using forced entry and a hierarchical method. Linearity of the continuous variables with respect to the logit of the dependent variable was assessed by the Box‐Tidwell procedure, and a Bonferroni correction was applied using all terms in the model to assess its statistical significance. Multicollinearity was excluded after checking tolerance and variance inflation factor statistics and the proportion of the variance of each predictor's b value attributed to each eigenvalue. The ability of the model to discriminate between outcome categories was investigated in more detail by elaborating the ROC curve. This analysis was performed for LT4 × week/BMR ratio vs deiodinase activity on the basis of the regression outputs. Youden's best cut‐off was also calculated, and the greater values were chosen to balance the better sensitivity and specificity for the studied variable.

## 3 RESULTS

### 3.1 Patient evaluation according to GD

#### 3.1.1 Univariate analysis

Structure parameter influence assay (SPINA) revealed that GD was reduced in 50/160 (31.2%) of the thyroidectomized patients (Table 1).

**TABLE 1:** Characteristics of the 160 athyreotic patients treated with L‐T4

Age (years)	44.6 (13.9)
Sex (F/M)	117/43
Weight (kg)	73.1 (18.3)
Height (cm)	163.6 (12.2)
BMI (kg/Height^2^)	27.1 (6.1)
TSH (mU/L)	1.6 (0.4–2.9)
FT4 (pmol/L)	14.1 (11.6–21.9)
FT3 (pmol/L)	4.1 (2.1–5.4)
FT3/FT4ratio	0.25 (0.05)
BMR (Kcal/24 h)	1419.1 (265.5)
LT‐4 × week/BMRr (μg)	0.6 (0.4–1.4)
LT‐4 × week (μg)	835.7 (238.7)
GD (nmol/s)	24.1 (12.0–40.0)
GD < 21 nmol/s (*n*/%)	50/31.2
TSHI	2.0 (0.0–3.9)

*Note*: Data are expressed as mean and standard deviation or median and range between the brackets.

Abbreviations: BMR, basal metabolic rate; GD, calculated deiodinases activity; LT4 × week, LT‐4 cumulative weekly dose; LT4 × week/BMR, LT‐4 cumulative weekly dose/basal metabolic rate; TSHI, TSH index.

Patients were divided into two groups according to normal (≥21 nmol/s) or low (<21 nmol/s) GD. Sex, age, BMI and BMR were not different between the two groups (Table 2). Univariate analysis revealed that FT3 and the FT3/FT4 ratio were significantly reduced in patients with low GD compared to patients with normal GD (*p* < .004–.0001). However, in low GD TSHI, FT4, LT‐4 weekly cumulative dose (LT‐4 × week) and the ratio between LT‐4 weekly cumulative dose and basal metabolic rate (LT‐4 × week/BMR) were significantly increased (*p* < .0001) (Table 2).

**TABLE 2:** Characteristics of the 160 according to calculated deiodination activity (GD)

	GD activity <21 nmol/s (*n* = 50)	GD activity ≥21 nmol/s (*n* = 110)	*p*
Age (years)	42.9 (17.3)	45.2 (12.1)	.6
Sex (F/M)	34/16	83/27	.2
Weight (kg)	73.3 (20.2)	73.1 (17.5)	.9
Height (cm)	162.9 (16.9)	163.9 (9.5)	.7
BMI (kg/Height^2^)	26.8 (6.7)	27.1 (5.6)	.6
TSH (mU/L)^a^	0.8 (0.1)	1.0 (0.1)	.4
FT4 (pmol/L)^a^	18.1 (2.0)	14.2 (2.6)	.0001
FT3 (pmol/L)^a^	3.7 (0.1)	4.3 (0.0)	.0001
FT3/FT4 ratio^a^	0.20 (0.05)	0.30 (0.05)	.0001
BMR (Kcal/24 h)^a^	1435.1 (40.3)	1409.8 (24.7)	.4
LT‐4 × week/BMRr (μg/BMR)	0.6 (0.2)	0.5 (0.1)	.01
LT‐4 × week (μg)	910.2 (259.2)	802.5 (222.3)	.006
GD (nmol/s)	18.3 (0.3)	26.6 (0.3)	.0001
TSHI^a^	2.2 (0.1)	1.7 (0.1)	.004

^a^ Data are expressed as median and standard error between the brackets.

#### 3.1.2 Binary logistic regression analysis and ROC curve

Variables reaching statistical significance by univariate analysis were then analysed by binary logistic regression analysis models. LT‐4 × week/BMR was independently and inversely related to GD [*B* = −3.88, wald = 7.6, *R* = 0.021 (0.001–0.329; 95% confidence interval (CI)), *p* = .006], FT3 levels were directly and independently related to GD [*B* = 2.81, wald = 25.1, *R* = 17.4 (5.6–53.4, 95% CI) *p* = .0001]. In contrast, BMR, BMI, body weight, TSH and FT4 were not independently related to GD. To evaluate the effect of LT4 × week/BMR on GD, we used a classic receiver operating characteristic (ROC) model that was very well validated by the study of area under the curve (AUC) = 0.81 ± 0.073 (0.66–0.95, 95% CI, *p* = .001). To better define the cut‐off of LT‐4 dose beyond which GD was reduced, we researched the best cut‐off of Youden's statistic (YS). YS = 60 indicates that LT‐4 × week/BMR > 0.56 mcg × week/kcal is a good predictor of suppressed GD with sensitivity = 83% and specificity = 77% (e.g. a total of 144 mcg of LT‐4 daily dose reduces GD in patients with 1800 kcal/die estimated BMR).

### 3.2 Patient evaluation according to BMI

#### 3.2.1 Linear regression, complete or partial bivariate analysis with calculation of Pearson coefficient

FT3 and FT4 were increased in obese patients compared with nonobese patients (*p* = .07 and *p* = .01, respectively), while GD and LT‐4 × week/BMR were similar in the two groups (Table 3).

**TABLE 3:** Characteristics of 160 patients according to BMI

	Non‐obese (*n* = 120)	BMI ≥30 < 35 (*n* = 20)	BMI ≥35 (*n* = 20)	*p*
Sex m/f	28/92	8/12	7/13	
Age (years)	43.4 (14.5)	46.7 (11.5)	48.3 (11.1)	.1
Weight (kg)	65.5 (12.2)	89.3 (10.8)	101.6 (16.4)	.00
Height (cm)	163.1 (12.7)	167.9 (10.5)	160.9 (8.8)	.4
BMI (weight/[height]^2^)	24.3 (3.2)	31.6 (1.3)	39.7 (4.9)	.00
TSH (mU/L)	0.8 (0.05)	1.0 (0.2)	1.3 (0.2)	.6
TSHI	1.7 (0.8)	2.1 (0.7)	2.2 (0.2)	.1
FT4 (pmol/L)	15.6 (2.4)	16.7 (3.2)	17.7 (4.2)	.01
FT3 (pmol/L)	3.9 (0.6)	4.1 (0.6)	4.3 (0.5)	.07
FT3/FT4 ratio	0.25 (0.04)	0.24 (0.05)	0.24 (0.05)	.1
BMR	1336.8 (216.6)	1633.4 (254.8)	1688.1 (258.4)	.00
GD (nmol/s)	24.1 (4.8)	23.7 (5.5)	24.4 (6.1)	.2
GD < 21 (nmol/s) *n*/%	36/30	7/35	7/35	.3
Lt4 × week/BMR	0.6 (0.1)	0.6 (0.1)	0.6 (0.1)	.3
Lt4 × week (μg)	779.2 (214.1)	1006.9 (270.3)	1002.0 (173.1)	.00

*Note*: Data are expressed as media and SD between the brackets.

**FIGURE 2:**
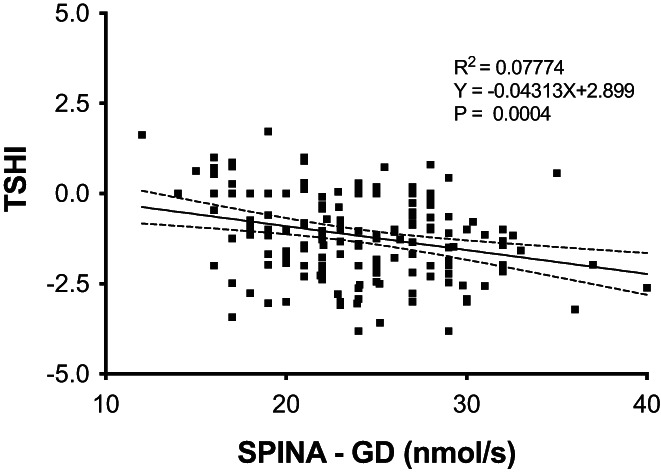
Linear correlation of SPINA GD (nmol/s) with TSHI

**FIGURE 3:**
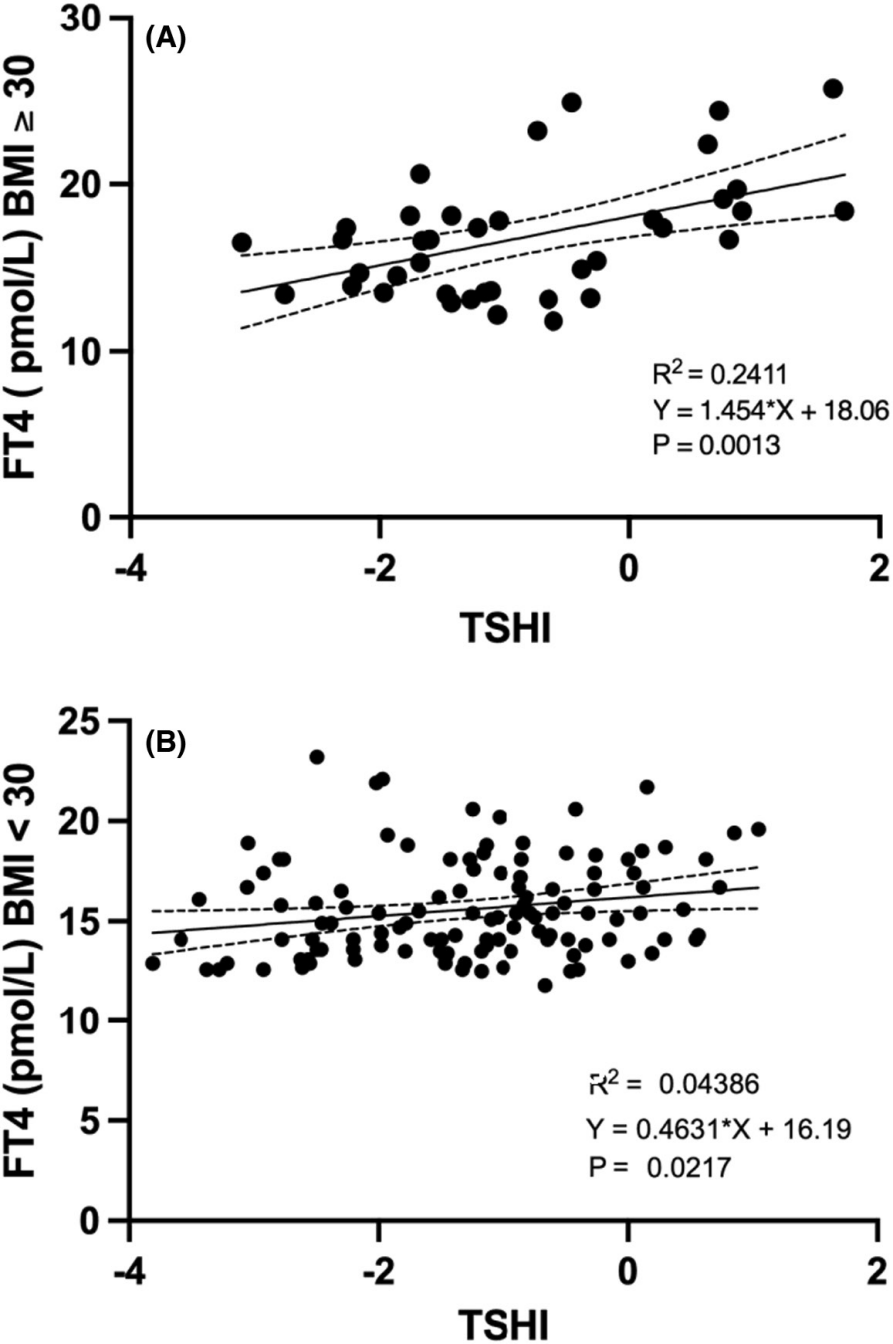
(A and B) Correlation between TSHI and FT4 in obese and non‐obese athyreotic patients

Partial bivariate analysis revealed that FT4 levels were positively related to BMI and negatively related to BMR after subtraction of the BMI effect: *p* = .01 and *p* = .02. Pituitary thyreotropic activity, evaluated by TSHI, was positively related to BMI and LT‐4 × week/BMR: *R* = 0.13, *p* = .05, *R* = 0.14, *p* = .03 and inversely related to GD: *p* = .0004 (Figure 2). FT4 levels were positively related to TSHI in both obese and nonobese patients. In obese patients, the FT4 to TSHI increment was 3.3 times greater than the increment in nonobese patients: 0.1% versus 0.03%, *R* ^2^ = .24, *p* = .001 versus *R* ^2^ = .04, *p* = .02 (BMI ≥ 30 vs. BMI < 30) (Figure 3).

**FIGURE 4:**
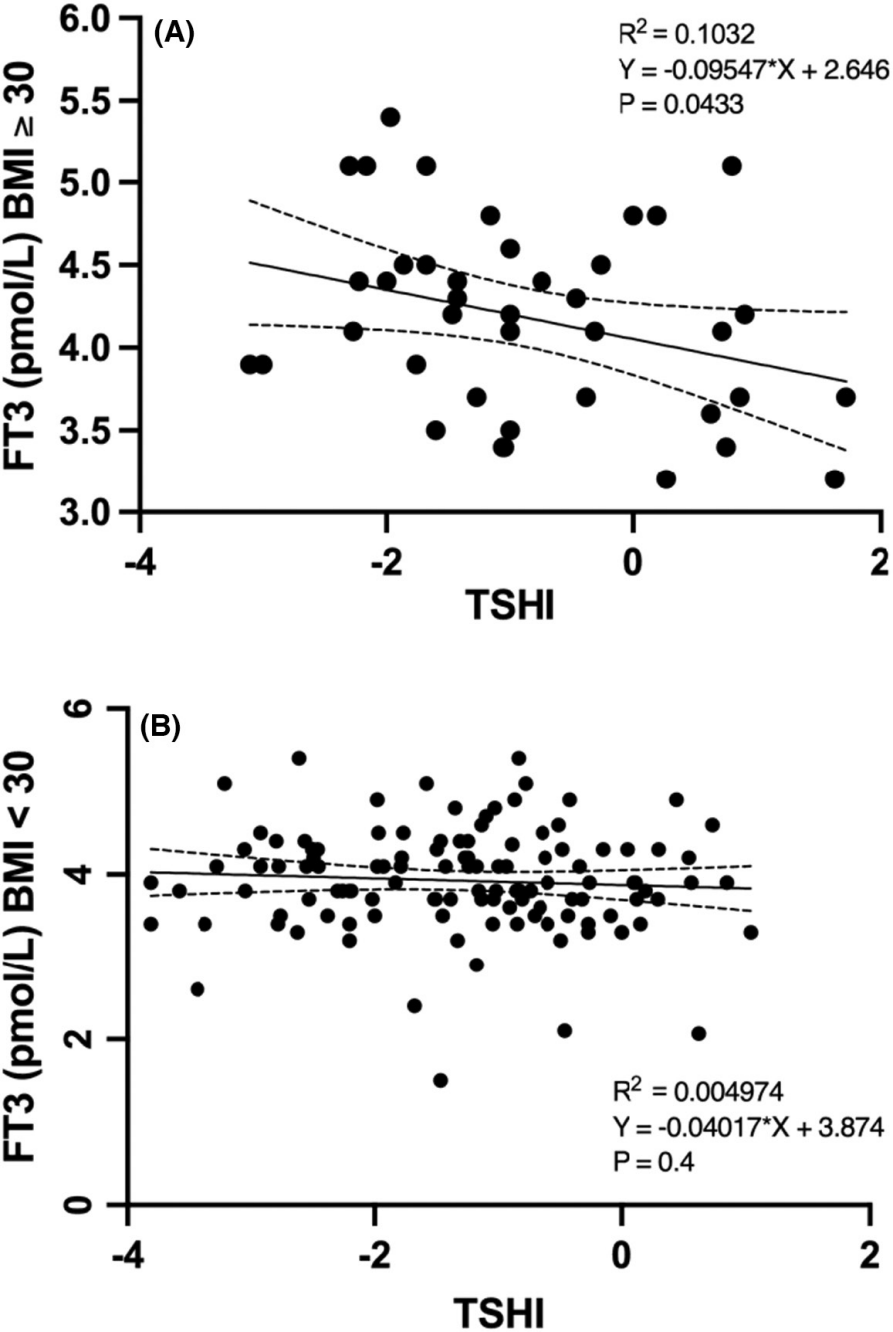
(A and B) Correlation between TSHI and FT3 in obese and non‐obese athyreotic patients

In obese patients (*n* = 40), FT3 levels were inversely related to TSHI; a TSHI increment of 1 unit was related to an FT3 decrement of 0.095%: *R* ^2^ = .1; *p* = .045. In contrast, FT3 levels were not related to TSHI variations in nonobese patients: *R* ^2^ = .002, *p* = .58 (Figure 4).

These data confirm that the feedback sensitivity of thyroid hormones with the pituitary is significantly different in obese and nonobese patients.

## 4 DISCUSSION

Several lines of evidence indicate that hypothyroid patients under levothyroxine replacement therapy may present impaired T3 production and a reduced T3/T4 ratio.^13^ The T3 pool derived from intrathyroidal conversion is absent and fails to maintain normal FT3 levels. As a consequence, their peripheral tissues may be underexposed to circulating T3. Our previous data indicate that 29.6% of levothyroxine‐treated athyreotic patients have a reduced FT3/FT4 ratio, and this percentage may progressively increase with increasing replacement levothyroxine dose.^17^ These changes may be due to an imbalance between central and peripheral deiodinase activity that may disrupt thyroid hormone homeostasis in this subset of hypothyroid patients.^12^, ^13^, ^14^ In our study, we evaluated the total deiodinase activity (GD) by the SPINA cybernetic model.^15^, ^16^ We found that our athyreotic patients with impaired GD received a larger dose of LT‐4 and had increased FT4 and TSHI levels, while the FT3/FT4 ratio and FT3 levels were reduced (all <0.0001). GD was reduced in 31.2% of study patients, confirming our previous report since GD is well correlated with the FT3/FT4 ratio^17^ (Table 2). To better evaluate the interplay between GD, BMR and LT‐4 weekly cumulative dose (LT‐4 × week), we evaluated the ratio between LT‐4 × week and basal metabolic rate (LT‐4 × week/BMR) calculated by the formula of Mifflin St.‐Jeor. By this tool largely used to evaluate BMR in obese patients,^18^, ^19^ we demonstrated that total GD activity was independently and inversely related to LT‐4 × week/BMR. According to this view, we analysed a subgroup of 45 patients with a stable LT‐4 dose, caloric intake and level of thyroid hormones for almost six months, and we found that a LT‐4 × week/BMR value of 0.56 mcg × week/Kcal can predict the impairment of GD (<21 nmol/s) with good sensitivity and specificity (*p* = .01). To our knowledge, this is a new finding with a possible clinical implication in athyreotic patients receiving LT‐4 substitutive therapy. Interestingly, estimated BMR, BMI, age and sex were similar between the patients with normal or reduced GD, suggesting that LT‐4 dose and FT3 production are the two independent stronger predictors of GD. Cross‐sectional and longitudinal studies comparing post‐ and presurgical levels of L‐T4 prove that higher L‐T4 doses are associated with the suppression of deiodinase activity.^16^ FT4 and FT3 were higher in our obese (BMI ≥ 30) than in nonobese patients (BMI < 30) (*p* = .01, *p* = .07), and TSHI was positively related to BMI and LT‐4 × week/BMR and inversely related to GD. However, GD and LT‐4 × week/BRM were not different between obese and nonobese patients, suggesting that BMI is not an independent determinant of GD. The pituitary thyrotropic activity, expressed by the relationship between TSHI and thyroid hormone levels, was different between nonobese and obese patients. TSHI suppression was constantly exerted by increasing levels of FT4 in nonobese patients, while this suppression was significantly attenuated at higher levels of FT4 in obese patients, suggesting increased hypothalamic–pituitary resistance in response to increased T4 levels. The increment of FT4 for each unit of TSHI increment was significantly higher in obese patients than in nonobese patients (*p* = .04) (Figure 3). However, in accordance with the FT4 results, increasing levels of FT3 constantly suppressed TSHI in nonobese patients, while this suppression was increased at increasing levels of FT3 in obese patients (Figure 4). This T4 to T3 shunt, in terms of TSHI suppression observed in obese patients, suggests a defective hypothalamic pituitary T4 to T3 conversion. Moreover, FT4 levels were positively related to BMI as well as to T4 dose but only partially and inversely related to BMR when BMI effect was subtracted (Pearson, *p* = .01). Considering that FT4 levels in athyreotic patients are entirely dependent on LT‐4 adsorbed dose and on the extent of T4 degradation,^17^ this finding unravels a role of BMR on the modulation of FT4 bioavailability both in nonobese and in obese patients, those with greater lean body mass that leads to increased BMR.^6^, ^7^, ^8^ Differently than some recent studies,^20^ we did not evidence a statistically significant correlation of GD with BMR, however differently than the others studies we evaluated patients athyreotic by total thyroidectomy and ^131^I ablation, this might contribute to increase the severity of suppression of the feedback loop and the ability to relay type 1 and type 2 allostatic load to T3 production. Moreover, we did not evaluate separately free fat mass and lean body mass. Under normal conditions, thyroid hormones and TSH are inversely correlated, while in patients with resistance to thyroid hormone, higher thyroid hormone levels correspond to high TSH levels due to a possible condition of resistance to FT4, such as in obese patients.^9^, ^10^, ^21^, ^22^ One study demonstrated that deiodinase ubiquitination was an important factor in restoring euthyroidism. Indeed, the ubiquitin proteasome system in the hypothalamus of obese mice fails to maintain adequate function. Hence, a defective function of the ubiquitin proteasome system, resulting in deiodinase imbalance, might play a major role in the regulation of the response to thyroid hormones in obese subjects.^9^, ^21^, ^22^, ^23^ Thyroid hormone action is modulated by the hypothalamic pituitary thyroid axis,^6^ and cell membrane transport, tissue deiodination and degradation and thyroid hormone metabolism in the liver may play an important role.^9^, ^22^, ^23^, ^24^ Metabolism of exogenous substrates in the liver occurs by enzymes that either modify and/or conjugate the functional groups to endogenous substrates to increase their solubility to be readily eliminated. Approximately half of obese subjects display several abnormalities in liver enzymatic activity due to steatosis.^25^, ^26^, ^27^ In particular, increasing BMI and thyroid hormone receptor β are inversely correlated with different stages of nonalcoholic fatty liver disease (NAFLD),^28^, ^29^ which, in turn, is related to decreased multidrug resistance protein (MRP2) activity in the liver. This condition is associated with alterations in the expression and function of enzymes and transporters resulting in an altered glucuronoconjugation of thyroid hormones.^23^ However, our study is descriptive and does not allow any direct evaluation of mechanistic insights related to T4 activation, degradation and stability.

## 5 CONCLUSIONS

Approximately one‐third of athyreotic patients under LT‐4 replacement therapy have reduced GD. GD activity is inversely and independently related to LT‐4 dose and FT3 levels. We found that an LT‐4 weekly cumulative dose of 0.56 mcg/kcal was an independent predictor of reduced GD, while sex, age, BMI or BMR were not. FT4 levels are higher in athyreotic obese patients, who therefore appear more resistant to LT‐4 replacement therapy. Indeed, FT4 is positively related to BMI and inversely related to BMR, which, in turn, negatively modulates the FT4 increment, especially in patients with elevated body lean mass. Other metabolic pathways both centrally and perimetrically might be involved in FT4 and FT3 degradation.

## AUTHOR CONTRIBUTIONS

**Pasqualino Malandrino:** Data curation (equal); validation (equal). **Marco Russo:** Data curation (equal); validation (equal). **Dario Tumino:** Data curation (equal); validation (equal); visualization (equal). **Tommaso Piticchio:** Data curation (equal); formal analysis (equal); visualization (equal). **Adriano Naselli:** Data curation (equal); formal analysis (equal); software (lead); supervision (equal); writing – review and editing (equal). **Valentina Rapicavoli:** Data curation (equal); resources (equal). **Antonino Belfiore:** Conceptualization (equal); funding acquisition (equal); methodology (equal); project administration (equal); resources (equal); supervision (equal); validation (equal); visualization (equal); writing – review and editing (equal). **Francesco Frasca:** Conceptualization (equal); funding acquisition (equal); investigation (lead); methodology (equal); project administration (equal); resources (equal); supervision (equal); validation (equal); visualization (equal); writing – review and editing (equal). **Rosario Le Moli:** Conceptualization (equal); data curation (equal); formal analysis (equal); investigation (equal); methodology (equal); project administration (equal); software (equal); supervision (equal); validation (equal); visualization (equal); writing – original draft (equal); writing – review and editing (equal).

## CONFLICT OF INTEREST

The authors declare no conflict of interest.

## INSTITUTIONAL REVIEW BOARD STATEMENT

The studies involving human participants were reviewed and approved by Ethics Committee Garibaldi Nesima Hospital ‐ Catania.

## INFORMED CONSENT

Written informed consent for participation was not required for this study in accordance with the national legislation and the institutional requirements.

## Data Availability

The data presented in this study are available on request from the corresponding author.
